# A Particular Focus on the Prevalence of *α*- and *β*-Thalassemia in Western Sicilian Population from Trapani Province in the COVID-19 Era

**DOI:** 10.3390/ijms24054809

**Published:** 2023-03-02

**Authors:** Rossella Daidone, Antonella Carollo, Maria Patrizia Perricone, Renato Messina, Carmela Rita Balistreri

**Affiliations:** 1 Transfusion Medicine Unit, San Antonio Abate Hospital, Cosenza Street, n.82, 91016 Erice, Italy; 2 Pediatric and Thalassemia Unit, San Antonio Abate Hospital, Cosenza Street, n.82, 91016 Erice, Italy; 3 Cellular and Molecular Laboratory, Department of Biomedicine, Neuroscience and Advanced Diagnostics (Bi.N.D.), University of Palermo, 90134 Palermo, Italy

**Keywords:** thalassemia, *α-* and *β-globin* genes defects, Trapani population, genotype, phenotype in the diagnosis

## Abstract

Thalassemia is a Mendelian inherited blood disease caused by *α-* and *β-globin* gene mutations, known as one of the major health problems of Mediterranean populations. Here, we examined the distribution of *α-* and *β-globin* gene defects in the Trapani province population. A total of 2,401 individuals from Trapani province were enrolled from January 2007 to December 2021, and routine methodologies were used for detecting the *α-* and *β-globin* genic variants. Appropriate analysis was also performed. Eight mutations in the *α globin* gene showed the highest frequency in the sample studied; three of these genetic variants represented the 94% of the total α-thalassemia mutations observed, including the −α3.7 deletion (76%), and the tripling of the α gene (12%) and of the α2 point mutation IVS1-5nt (6%). For the *β-globin* gene, 12 mutations were detected, six of which constituted 83.4% of the total number of β-thalassemia defects observed, including *codon β039* (38%), *IVS1.6 T > C* (15.6%), *IVS1.110 G >* A (11.8%), *IVS1.1 G > A* (11%), *IVS2.745 C > G* (4%), and *IVS2.1 G > A* (3%). However, the comparison of these frequencies with those detected in the population of other Sicilian provinces did not demonstrate significant differences, but it contrarily revealed a similitude. The data presented in this retrospective study help provide a picture of the prevalence of defects on the *α* and *β-globin* genes in the province of Trapani. The identification of mutations in *globin* genes in a population is required for carrier screening and for an accurate prenatal diagnosis. It is important and necessary to continue promoting public awareness campaigns and screening programs.

## 1. Introduction

Thalassemia is a Mendelian inherited blood disease caused by *α-* and *β-globin* gene mutations, known as one of the major health problems of Mediterranean populations [1,2,3]. Due to the phenomenon of immigration, the area of diffusion is, however, growing towards the more industrialized countries of the world, assuming a global character [4]. Consequently, a significant number of cases has begun to emerge in diverse nationalities, even devoid of this type of patient, also linked to inadequate prevention [5,6]. This trend has been particularly documented during the COVID-19 pandemic because the highest peak of mortality has also been estimated in patients with undiagnosed thalassemia, whose prevalence rates were assent or insignificant in the involved populations [7,8,9,10]. Sicily, and particularly the Trapani province, constitutes one of the major sites of immigration, and one of the Sicilian provinces with the highest number of COVID-19 cases (see this istat link: https://statistichecoronavirus.it/coronavirus-italia/coronavirus-sicilia/coronavirus-trapani/; 18 February 2023) [11]. However, the existence of a relationship between the above-mentioned estimations and the higher frequencies of defects in the *α-* and *β-globin* genes in the Trapani population has not been reported so far [12,13]. To shed light on the genetic, geographic, and ethnic factors which could have contributed, and which are contributing to the heterogenous incidence and prevalence of the COVID-19 pandemic in the diverse populations of the provinces of our island, we wanted, in this retrospective study, to provide a little piece of such a complex puzzle by detecting the incidence of the different beta-globin gene mutations in the Sicilian population and the distribution of *α-* and *β-globin* gene defects in a total of 2401 individuals from Trapani province enrolled from January 2007 to December 2021, comparing the data with those of other Sicilian provinces. In addition, we determined the correlation between the genetic and phenotypic features in the carriers of *α-* and *β-globin* genetic variants.

## 2. Results

**Figure 1:**
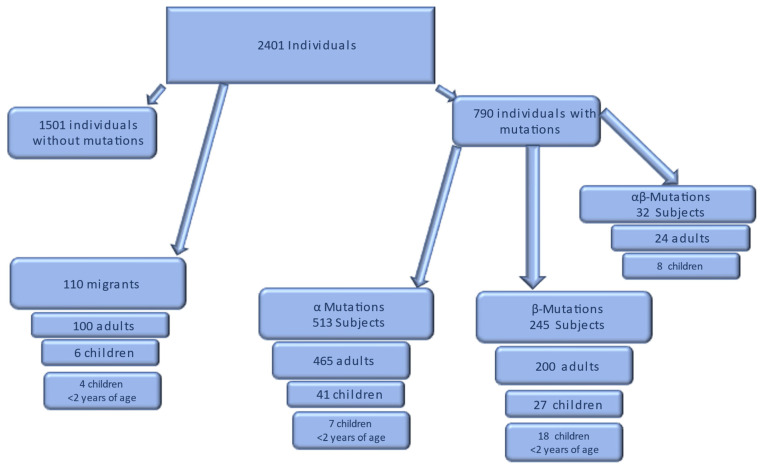
Schematic representation of study’s sample.

The molecular investigation and its analysis first allowed to exclude in such study 1501 subjects who did not have any mutation in the *α* or *β globin* gene or both genes. Likewise, the 110 African individuals, (4.6%) identified by considering the last name of patients and their nationality of origin, were also not included in the study (see Figure 1). However, we reported the data relating to the globin gene variants detected in this last group; the number of cases observed, and frequencies are reported in Table 1.

**Figure 2:**
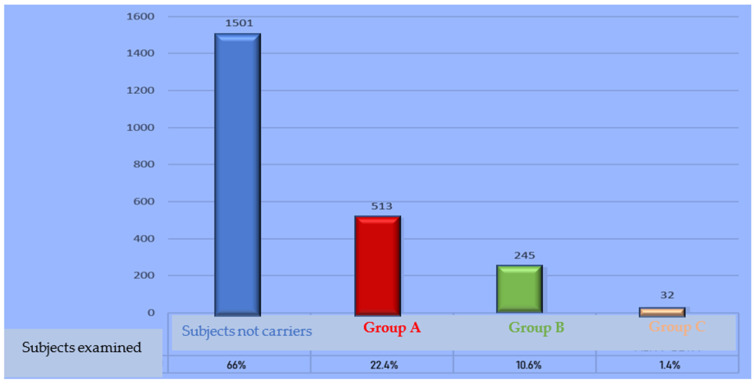
Populations grouped in carriers or not and for mutations showed.

In addition, this evaluation evidenced that 34% of the population examined, equal to 790 subjects born in Trapani province, were carriers of a mutation in the *α* or *β globin* gene or both genes (see Figure 1). Consequently, the 790 subjects were divided into three groups: (1) Group A: subjects with mutations in the *α* genes (22.4%), (2) Group B: subjects with mutations in the *β* genes (10.6%), and (3) Group C: subjects with mutations in both genes (1.4%). For each group, the frequency of the individual mutations detected was calculated. The number of cases observed for each group and the relative percentages are reported in Figure 2.

The study sample also included 110 (4%) (see Figure 1) foreign subjects residing in the province of Trapani. Of these subjects, 59 (54%) did not have a mutation, and 51 (46%) had a mutation in the *α-* or *β-globin* gene or both. The most frequent mutation of the *α* gene was the *-α3.7 deletion* (51%); the most common mutation of the *β* gene was the *A > T substitution on codon 6* with formation of the haemoglobin variant HbS (6%). The most frequent *α + β* genotype was determined by the *deletion -α3.7/codon 6 A > T HbS* (16%).

Five of the patients examined were affected by sickle cell anaemia and one subject was affected by micro-drepanocytosis, a condition characterized by compound heterozygosity.

### 2.1. Group A

**Figure 3:**
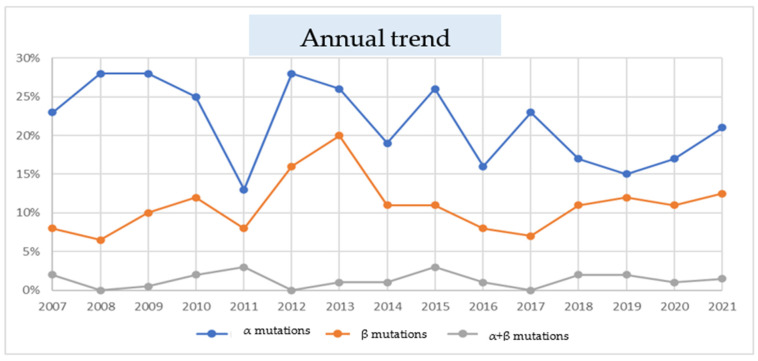
Annual trend of the examined mutations.

Group A included patients with one or more mutations in the *α* globin genes. These subjects constituted 65% (number of cases observed, 513; see Figure 3) of the total number of patients with such mutations, and 22.4% of subjects when compared to the total number of individuals examined (2291 subjects born in Trapani province). From 2007 to 2021, 9 mutations affecting the *α-globin* gene were observed, including the most frequent mutation, which is the *-α3.7 deletion* (76%), followed by the triplication of the *α* gene (12%), the *α2 point mutation IVS1 -5nt* (6%), and the *-α4.2 deletion* (1%). The other mutations identified show a <1% frequency. A total of 2% of the subjects examined had the *-α3.7 deletion* in homozygous condition. In five observed cases, subjects were doubly heterozygous for two of the *α* mutations. The data relating to the number of cases observed for each single mutation and the percentages are shown in Table 2.

### 2.2. Group B

Group B included patients with one or more mutations in the *β globin* genes. These subjects constituted 31% (number of cases observed, 245; see Figure 2) of the total number of patients with mutations, and 10.6% of the total number of examined individuals (2291 born in Trapani province, see Figure 1). From 2007 to 2021, 12 mutations in the *β* globin gene were observed. The *β039 codon mutation* is the most common (38%); the second is *IVS1.6 T > C* (15%), followed by *IVS1.110 G > A* (12%) and *IVS1.1 G > A* (11%). Six mutations showed a frequency lower than or equal to 5%: codon 6 A > T with formation of the haemoglobin variant HbS (5%), *IVS2.745 C > G* (4%), *IVS2.1 G > A* (3%), *codon 6 -A* (2.4%), *−101 C > T* (1.6%), and *−87 C > G* (1.6%). The remaining two mutations identified have a very low frequency (<1%). Considering the genotypes, five different ones were observed among patients with intermedia thalassemia, and two among patients with thalassemia major. Genotypes attributable to thalassemia intermedia included homozygosity for *IVS1.6 T > C* and compound heterozygosity for a mild *−101 C > T mutation* or the haemoglobin variant Hb C. Among individuals with thalassemia major, the genotype most frequent is represented by compound heterozygosity *β0/β+ codon39/IVS1.110 G > A* (1.2%). The data relating to the number of cases observed for each mutation and the relative percentages are shown in Table 2.

### 2.3. Group C

Group C included patients with mutations in the *α* and *β globin* genes. These subjects constituted 4% (number of cases observed, 32; see Figure 3) of the total number of patients with the mutation, and 1.4% of the total number of individuals examined (2291 born in Trapani province, see Figure 3). From 2007 to 2021, 13 different *α + β* genotypes were observed. The most frequent genotype is represented by the *α/codon 39 C > T triplication* (19%), the second by the deletion *-α3.7/codon 39 C > T* (16%), followed by the -*α3.7/codon 6 A > deletion T* (HbS) (16%), and the *deletion -α3.7/IVS1.6 T > C* (16%). The genotypes determined by the *deletion -α3.7/IVS1.1 G > A* and the triplication *α/IVS2.745 C > G* are observed in a percentage of 6%. The other genotypes were observed with a frequency of 3%. Two of the subjects, in addition to the mutation on the *α* globin gene, have a double heterozygosity with mutations affecting both *β* genes. The data relating to the number of cases observed for the different genotypes and the relative percentages are shown in Table 2.

### 2.4. Annual Trend of Mutations Examined

The annual trend of mutations on the *α* and *β globin* genes was evaluated by calculating the percentage of subjects carrying the specific mutations with respect to the total number of subjects examined for each single year, from 2007 to 2021. The percentage of subjects with mutations in the *α globin* genes showed a decreasing trend from 2007 to 2021, with two major peaks of 28% observed in the years 2008 and 2009, and a minimum of 13% in 2011. The percentage of subjects with mutations in β globin genes showed, overall, a reduced trend with a maximum peak of 20% in 2013 and a minimum of 6.5% in 2008, and with values which did not exceed 5%. Particular attention should be paid to the years 2020 and 2021, in which the number of subjects subjected to molecular investigation for the thalassemia genotype was 88 and 56, respectively, as due to the ongoing pandemic from Covid-19 no HPLC screenings were carried out in schools, and this, consequently, had an impact on the number of subjects for which genotype analysis may be requested. The most frequent mutation in the *α* gene in all years was the *-α3.7 deletion*. The most frequent mutation of the *β* gene in the single years examined was the *nonsense mutation of codon 39*, except for the year 2010, in which the most frequently detected β mutation was *IVS1.6 T > C*. The trend illustrated is shown in Figure 3.

### 2.5. Correlation between Genotypes and Phenotypes

To evaluate the correlation between a given genotype and the parameters related to haemoglobin, haemoglobin and blood red cell parameters were detected. Data obtained were divided among the cases examined, according to their age (by differentiating adults from children aged between 3 and 12 years, because the blood parameters differ, and especially the values of haemoglobin and mean cell volume: MCV). 212 subjects were excluded because of a lack of relative information on haemoglobin and/or blood parameters, 36 of which were less than 2 years old at the time of the molecular analysis; in younger children the examination of the HPLC haemoglobin testing is not performed because it is unreliable due to the rise of the percentage of Hb-F, reaching adult levels (<1%) by 2 years of age. Subjects with a haemoglobin variant affecting the α or β globin chain and subjects with diseases are considered separately to avoid errors in statistical analysis.

Subjects with mutations in the *α globin* gene had, overall, normal or slightly decreased HbA2 values, and normal Hb-F. As for the blood red cell parameters, a slight decrease in MCV value and greater reduction in MCH levels constituted a greater discriminant in *α*-thalassemia carriers. A more marked decline in MCV values (<70 fL) and MCH (<24 pg) was observed in subjects with *α0-*thalassemia and, therefore, in the presence of *-α3.7 deletion* homozygous, --MED, and double heterozygous for the *-α3.7 deletion* and *α2 point mutation IVS1 -5nt*. Among carriers of the -*α3.7 deletion*, one subject co-inherited the haemoglobin Hb Lepore variant, which in HPLC coelutes in correspondence with HbA2 and, therefore, the values of this fraction of Hb were indeterminate. A double is observed in one heterozygous patient for the -*α3.7 deletion* and the *--MED deletion*, with HbA2 values of 0.7% and HbH (β4 tetramer) values of 7.9%. Patients with the triplication of the *α* gene deserve special attention, showing reduced MCH values accompanied by normal or borderline HbA2 values, which make their identification more insidious. Children with mutations in the *α* globin gene had normal HbA2 values and normal or slightly increased values of Hb-F. MCV and MCH values were in general normal; a decrease was only observed in the carrier of the *-α3.7 deletion* in homozygosity (*α0-thalassaemia trait*). One of children with the *-α3.7 deletion* had the haemoglobin Hb D-Los Angeles variant (or Hb D-Pujab) and was considered separately. Normal RDW-C% values were detected in all the patients examined; this parameter allows to discriminate the carriers of thalassemia from a state of iron deficiency. The number of subjects observed for each mutation and the values relative to the mean ± deviation standards are shown in Table 3.

Individuals with mutations in the *β globin* genes generally had increased values of HbA2 and hypochromic microcytic anaemia, with differences depending on the type of inherited mutation. Individuals with the *β+ mutation* showed greater variability in phenotypic characteristics, with slightly increased HbA2 values in carriers of *mild or silent β mutations*, *−101 C > T* (mean HbA2 values 3.75% ± 0.24) and *IVSI.6 T > C* (mean HbA2 values 3.81% ± 0.46). The classic β thalassemia trait phenotype with increased HbA2, decreased MCV values, MCH, and anaemia was observed, however, in carriers of the *β+ mutations* affecting the sites of the splicing of β-globin pre-mRNA (*IVS1.110 G > A* and *IVS2.745 C > G*). The bearers of mutation in the promoter of the *β* gene, the *−87 C > G*, in addition to the normal characteristics of the β trait thalassemia showed an increase in Hb-F, with maximum values of 4.60% found in adults and 8.70% found in children. Data relating to the number of observed cases, mean, and standard deviation are shown in Table 4. More homogeneous phenotypic characteristics are observed in subjects with *β0 mutations*, because of the absence of β-globin chain production by the mutant allele. The subjects had increased HbA2 values (mean values >4.9%), decreased MCV values (<65 fL), reduced MCH values (<20 pg), and anaemia (mean Hb values of 10 g/dL). The data relating to the number of observed cases, mean, and standard deviation are shown in Table 4.

Among the subjects examined, 10 were carriers of the HbS haemoglobin variant; they showed normal phenotypic features, sometimes with slightly increased HbA2 values. The data relating to the number of cases observed, the mean, and standard deviation are shown in Table 5. Unfortunately, no other clinical data, such as their complications (e.g., splenomegaly), were available.

Co-inheritance of the *-α3.7 deletion* and a marked *β0 or β+ mutation* determines the typical phenotype of the β-thalassemia trait, with increased values of HbA2, and MCV and MCH reduced. In carriers of the *-α3.7 deletion* and of the *mild β+ mutation (IVS1.6 T > C),* there were slightly increased HbA2 values, and decreased MCV and MCH values. A subject that was a carrier of the *-α3.7 deletion* and of the haemoglobin variant HbS showed slightly increased HbA2 values and a slight decrease in MCH. A subject that was a homozygous carrier of the *-α3.7 deletion* and the *β+ IVS2.745 C > G mutation* showed normal HbA2 values and reduced MCV and MCH values. In the co-inheritance of *α-tripling* and a *β0 or β+ mutation* there was an increased imbalance of globin chains with an excess of α chains. Most subjects with high HbA2 values (mean values >5%), low MCV, MCH, and anaemia developed a clinical phenotype of intermediate severity over time. The number of observed cases, the values relating to the mean, and standard deviation are shown in Table 6.

Finally, we also observed that 13 had a picture consistent with thalassemia intermedia or thalassemia major. Six different ones were observed among patients with thalassemia intermedia genotypes, including homozygosity for the *IVS1.6 T > C* mutation and compound heterozygosity for the slight mutation at the level of the *−101 C > T* promoter or for the haemoglobin Hb C variant. The presence of a slight thalassemia defect explained the minor severity of the clinical picture. Among the cases with thalassemia major, the most common genotype is compound heterozygosity for codon *39/IVS1.110 G > A* mutations. Based on the genotype, it is, however, possible to make only a prediction of the phenotype. The assignment of the severity and form of thalassemia, as intermedia or major form, certainly depends on measuring red blood cell indices, haemoglobin analysis, and assessing the clinical severity of anaemia. Molecular genetic testing may be useful for predicting the clinical phenotype and enabling pre-symptomatic diagnosis of at-risk family members and prenatal diagnosis [6].

## 3. Discussion

The hemoglobinopathies represent the most common monogenic disorders in humans, and comprise the thalassemia disorder, characterized by defective globin chain synthesis leading to chronic haemolytic anaemia [1,2,3]. Thalassemia is a group of hereditary disorders of haemoglobin synthesis that causes the absence or reduced production of globin chains [4]. An *α* cluster or not is defined as physiological when it produces the right amounts of chains in equal number between the two types and they have normal affinity for each other, so that no excess free chains are left. On the other hand, the cluster in which a defect causing a deficit, total, or partial synthesis of a chain (mainly α or β) to be established is defined as “thalassaemia” [4]. The study of the structure of genes has made it possible to highlight many of the structural alterations that are at the origin of thalassemia by confirming their heterogeneity both in molecular defects as well as in biochemical and phenotypic aspects. The mutations responsible for α-thalassemia are more than 120, and frequently are deletions in one or both *α* genes. Less point mutations affecting, above all, the *α2* gene are frequent. The identified mutations in the *β-globin* gene are more than 200, and include point mutations, single base substitutions, small insertions, or deletions within the gene or its flanking sequences [14,15,16].

Another typical feature of thalassemia is its higher prevalence and incidence in recognized geographic areas, including the Mediterranean Basin, Middle East, and Southeast Asia [4,5]. However, recent evidence underlines that thalassemia is assuming a worldwide distribution because of the migration of populations from high-prevalence territories to the Western World, a phenomenon recently increased because of the massive migration from Africa, the Middle East, and Asia to Europe [4,5]. Thus, thalassemia, and especially its transfusion-dependent thalassemia form, is becoming a challenging clinical condition, requiring both life-long care and follow-ups to be performed in specialized hubs and with the support of multidisciplinary teams of specialists. Such results in enormous healthcare costs. Consequently, it is necessary to have clear data on the frequencies of its mutations, and consequently prevalence of its forms, in precise geographic areas where such a disorder was inexistent and which were not ready to apply and develop clinically appropriate measures. About this aspect, it is imperative to be precise that the advances in diagnosis and treatment of thalassemia forms have intensely meliorated the prognosis and survival of affected patients, contributing to the possibility of having a normal lifespan at high quality [6]. However, such clinical improvements have not been uniformly adopted and applied to all patient populations, or in all the geographic regions, particularly in highly prevalent areas such as the Trapani province with its high rate of African migrants. Accordingly, the healthcare procedures in Western countries were not prepared until now to address the special needs of migrant thalassemia populations. Therefore, the management of patients with thalassemia is demanding.

In contribution to reducing this gap by providing recent epidemiologic data on the prevalence of the thalassemia mutations, as well as endorsing education, awareness, and support to the individuals affected and newly diagnosed, we conducted a retrospective study on a sample of 2401 subjects from the Trapani province enrolled from 2007 to 2021. On the other hand, the epidemiological data on the distribution of thalassemia mutations in the Sicilian islands goes back to those detected by Giambona team in 2011 [13]. The molecular analysis has led to evidence that eight mutations in the *α globin* gene showed the highest frequency in the sample studied: three of these defects represented 94% of the total α-thalassemia mutations observed, including *-α3.7 deletion* (76%), and the tripling of the α gene (12%) and of the α2 point mutation IVS1 -5nt (6%) (see data in Table 1). For the *β globin* gene, 12 mutations were detected, six of which constituted 83.4% of the total number of β-thalassemia defects observed, including *codon β039* (38%), *IVS1.6 T > C* (15.6%), *IVS1.110 G >* A (11.8%), *IVS1.1 G > A* (11%), *IVS2.745 C > G* (4%), *IVS2.1 G > A* (3%) (see data in Table 2). However, the comparison of these frequencies with those detected in the populations of other Sicilian provinces did not demonstrate significant differences, but contrarily revealed a similitude. The reason is linked to fact that, in Sicily, thalassemia is the most common hereditary blood disease and represents a serious public health problem [12,13,17]. In fact, a prevalence of 8% for α-thalassemia, 8% for β-thalassemia, 2.5% δ-globin gene defects, and 2% β-globin chain gene variants has been estimated in the entire population of Sicily [17]. The β-thalassemia defects in Sicily share the same set of mutations as other Mediterranean countries. The main factor behind their high frequency and distribution is the heterozygous advantage against Malaria infection; in Italy, in fact, the South and the major islands have always been considered former malarial geographical areas, and, here, subjects with thalassemia defects are particularly present [13]. The central location of Sicily and the natural resources have meant that in the past it was considered a strategic place for the commercial routes of the Mediterranean and a meeting place for Eastern and Western civilizations. The history of Sicily is, in fact, full of multiple invasions and occupations by different peoples, and this is the probable cause of the observed haemoglobin mutations and variants [18,19].

In a 2011 study conducted in Sicily by Giambona A. and colleagues [13], 30 mutations of β-thalassemia and 24 haemoglobin variants of the β chain were identified. The most common mutations of the *β gene* observed in almost all provinces are two, *codon β039* (31.85%) and *IVS1.110* (26.66%), except in the central areas (Enna and Caltanissetta) where a higher frequency of *IVS1.110* and *IVS1.1* mutations is found. The third most common mutation in Sicily is *IVS1.6* (14.62%), followed by *IVS1.1* (9.19%) and *IVS2.745* (5.87%). The latter is uniformly distributed in all Sicilian provinces except for Ragusa where it is present in high frequency (16.7%).^6^ As far as the α gene is concerned, the most frequent mutation in Sicily is the *-α3.7 deletion* (73.2%), even if a consistent number of subjects show triplication of the *α* gene [13].

In our study, the evolution of the frequency of mutations was also evaluated per single year, which showed a decreasing trend from 2007 to 2021 in a percentage of subjects carrying a mutation on the *α* and *β globin* genes, and an almost flat trend in the percentage of subjects who co-inherited a mutation on both globin genes (see Figure 3).

To evaluate the correlation between a given genotype and phenotypic characteristics, haemoglobin and red blood indices were evaluated in the subjects enrolled in our study with single mutations *α* or *β* or *both* genes [14,15,19,20]. Overall, subjects with *α gene* defects had normal or slightly decreased HbA2 values, a slight decrease in MCV, and a greater decrease in the MCH value which constituted the major discriminant. Subjects with α^0^-thalassemia showed a phenotype with a more marked reduction of MCV and MCH (see data reported in Table 3). The phenotypic features of the α-thalassemia trait may be confused with iron deficiency, as both manifest as hypochromic microcytic anaemia. Particular attention should be paid to the parameter RDW-C% [21,22,23], which allowed to distinguish the two conditions. Iron deficiency is accompanied by an increase in RDWC%, while in the thalassaemic trait this parameter has normal values.

Thalassemia *β* alleles may be classified, based on the production of β chains from the affected locus, as silent, mild, and severe [19,20]. In general, allele severity correlates with the degree of microcytosis and hypochromia, and carriers show greater heterogeneity in the haematological phenotype precisely based on the type of inherited mutation. As a rule, severe β defects determined by nonsense mutations, frameshifts, and mutations that abolish the process splicing all produce the haematological phenotype of β^0^-thalassemia, characterized by complete absence of β-chain production from the affected locus. The bearers showed the classic picture of the β-thalassemia trait with increased HbA2 values, reduced MCV and MCH values, and mild to moderate anaemia (see data reported in Table 4). On the other hand, mutations affecting the 5’ promoter region, consensus sequences flanking the invariant dinucleotides GT and AG, as well as mutations activating cryptic splice sites, produce a β^+^-thalassemia phenotype in which, depending on the type of mutation, a variable number of β-chains is produced from the affected locus. Carriers showed greater variability, with a phenotype ranging from the typical β-thalassemia trait to mild or silent conditions (see data reported in Table 4).

Patients with mutations in both globin genes have a haematological phenotype that depends on the type of inherited defect. The data reported in Table 6 indicate that the co-inheritance of a mild *α* defect (*deletion -α3.7*) in association with a *β^0^* mutation or *β+* determines the typical phenotype of the thalassaemic β trait. Marked haematological manifestations were seen in co-inheritance of α gene triplication and a *β^0^* or *β^+^* mutation, with increase in HbA2 and decrease in MCV, MCH, and anaemia. This agrees with the molecular basis of the inherited defects. In fact, subjects with triplication of the *α* gene have a greater imbalance of globin chains and develop over time a clinical picture compatible with thalassemia intermedia. In patients with heterozygous β-thalassemia, who present with a more severe than expected phenotype, interaction with triplicate α genes should therefore be considered. The potentially adverse effects of *α* gene tripling, when co-inherited with β-thalassemia, should be considered in the clinical care of these individuals, as they may develop transfusion needs or significant splenomegaly.

Thirteen patients affected by the disease with a clinical picture compatible with thalassemia intermedia or thalassemia major were observed. Subjects with thalassemia intermedia show a less serious clinical picture that does not require regular blood transfusions and is correlated with the presence in homozygous or compound heterozygotes of a mild or silent β mutation. Patients with thalassemia major show a serious, transfusion-dependent clinical picture that manifests itself from the first months of life and is linked to the inheritance of severe β mutations. Advances in molecular biology for DNA analysis have partially elucidated the relationship between genotype and phenotype. However, based on genotype alone, a prediction of the clinical picture may be made since each genotype is associated with a variable clinical outcome. Furthermore, the assignment of the severity and form of thalassemia, as intermedia or major form, certainly depends on diverse factors, i.e., on measuring red blood cell indices and haemoglobin analysis, and assessing the clinical severity of anaemia. Molecular genetic testing may be useful for predicting the clinical phenotype and enabling pre-symptomatic diagnosis of at-risk family members and prenatal diagnosis.

Identifying the molecular basis of thalassemia has greatly improved both the diagnosis and the clinical approach to the different forms of the disease, however, the presence of interactions with other genetic factors, known as modifier genes, still makes it difficult to establish the degree of relationship between genotype and phenotype. Consequently, it is imperative to adopt other technologies, such as those of the last generation, for improving the management of such disorders. The thalassemia International Federation (TIF), a global patient-driven umbrella federation with 232 member-associations in 62 countries [6], has tried and is trying to provide to all patients affected by thalassemia forms, or other hemoglobinopathies, an equal access to quality care in every region of the world by endorsing education, research, awareness, and support. One of TIF’s major activities is the development and dissemination of clinical practice guidelines for the management of these patients. They offer detailed information on the management of such patients and the clinical presentation, pathophysiology, diagnostic approach, and treatment of disease complications or other clinical entities that may occur in these patients, while also covering relevant psychosocial and organizational issues. However, it is also imperative to know the number of such patients and the mutations, and consequently the forms and the severity [6].

## 4. Materials and Methods 

### 4.1. Patients

Our retrospective sample of study was constituted of 2,401 individuals enrolled by the Transfusion Medicine Unit of San Antonio Abate Hospital, from January 2007 to December 2021. Inclusion criteria for the detection of carriers at the basic level were (i) low mean corpuscular volume (MCV) values (≤80 fL) with elevated Haemoglobin A2 (Hb A2) (≥3.6%), (ii) borderline Hb A2 values (between 3.3 and 3.5%), (iii) normal Hb A2 levels and elevated Haemoglobin F (Hb F) values, and (iv) MCV values <80 fL and HbA2 >3.5%. Of 2401 subjects investigated, 2197 (91%) were adult individuals, 160 (7%) were children with an age range from 3 to 12 years, and 44 (2%) were children with an age > of 2 years. In addition, 924 (38.5%) were men and 1,477 (61.5%) women. The sample also included 110 African individuals (4.6%), identified by considering the last name of patients and their nationality of origin. 

Of the autochthonous individuals, 1,501 (66%) did not have any *α-* and *β-globin* genes defects, while 790 individuals (34%) were carriers of one mutation in *α-* or *β-globin* genes or in both genes. Consequently, of 790 mutations carriers, three subgroups were identified: (I) individuals with mutations in *α* genes, (II) individuals with mutations in *β* genes, and (III) subjects with mutations in both genes. In each subgroup, the mutation with higher frequency was assessed, as well as the correlation between the genetic and phenotypic features in the carriers of *α-* and *β-globin* gene defects, and the prevalence of major or intermediate thalassemia (see Figure 1).

### 4.2. Genotyping

Haematological and biochemical data were determined in blood samples with EDTA anticoagulant and by using Beckman Coulter LH700 cytofluorimeter (Beckman Coulter s.r.l. Milan, Italy), and the haemoglobin parameters by using high performance liquid chromatography and Variant II, Dual kit Analyzer, Bio-Rad (Hercules, California, US). The molecular biology analysis was performed in DNA samples extracted from blood samples by using commercial Nuclear Laser Medicine (NLM; Settala, Milan, Italy) kits and standardized procedures. Allele-specific oligonucleotide hybridization (ASO) with radioactive probes, the commercial α-Globin StripAssay ^®^ e β-Globin StripAssay ^®^ (Vienna Lab Diagnostic) kits and ProfilBlot T48 Tecan revelation tool (Tecan Group, Männedorf, Switzerland) restriction enzyme analysis of amplified product (RE), amplification refractory mutation system (ARMS), and reverse dot blot (RDB) analysis, were carried out for direct detection of the most common mutations present in the Sicilian population. Radioactive sequencing and successively automatic sequencing were used to screen rare or unknown mutations by using methodologies previously published in the literature. Specific primers were used to identify deletional or recombinant defects present in Sicily (Sicilian δβ-deletion and Hb Lepore-Boston-Washington) [24,25]; analysis by multiplex ligation-dependent probe amplification (MLPA) was performed to screen other rare deletional defects [14,15]. Using this approach, all known mutations (globin.cse.psu.edu) of α- and β-globin genes were covered. Precisely, 21 mutations in the α-globin genes were determined: 3.7 single gene deletion, 4.2 single gene deletion, MED double gene deletion, SEA double gene deletion, THAI double gene deletion, FIL double gene deletion, 20.5 kb double gene deletion, anti-3.7 gene triplication, α1 cd 14 [TGG > TAG], α1 cd 59 [GGC > GAC] (Hb Adana), α2 int cd [ATG > ACG], α2 cd 19 [-G], α2 IVS1 [-5nt], α2 cd 59 [GGC > GAC], α2 cd 125 [CTG > CCG] (Hb Quong Sze), α2 cd 142 [TAA > CAA] (Hb Constant Spring), α2 cd 142 [TAA > AAA] (Hb Icaria), α2 cd 142 [TAA > TAT] (Hb Pakse), α2 cd 142 [TAA > TCA] (Hb Koya Dora), α2 poly A-1 [AATAAA-AATAAG], and α2 poly A-2 [AATAAA-AATGAA]. For the β-globin genes, 23 mutations were detected: −101 [C > T], −92 [C > T], −87 [C > G], −30 [T > A], codon 5 [-CT], codon 6 [G > A] Hb C, codon 6 [A > T] HbS, codon 6 [-A], codon 8 [-AA], codon 8/9 [+G], codon 30 [G > C], IVS 1.1 [G > A], IVS 1.2 [T > A], IVS 1.5 [G > C], IVS 1.6 [T > C], IVS 1.110 [G > A], IVS 1.116 [T > G], IVS 1–25 [25bp del], codon 39 [C > T], codon 44 [-C], IVS 2.1 [G > A], IVS 2.745 [C > G], and IVS 2.844 [C > G].

### 4.3. Statistical Analysis

The statistical analysis was performed by using SPSS software 20 version. The continuous haematological variables were expressed as media ± standard deviation, and the non-continuous were shown as percentages. The confidence interval (CI) at 95% was also detected, as well as the frequencies of mutations by using appropriate tests used routinely in our laboratories. 

## 5. Conclusions

The data presented in this retrospective study help to provide a picture of the prevalence of defects in the *α* and *β globin* genes in the population from Trapani province. The identification of mutations in *globin* genes in a population is required for carrier screening and for an accurate prenatal diagnosis, as well as to facilitate the current challenging management in children and adult people newly or not diagnosed. Certainly, the application of the TIF clinical practice guidelines for the management of these patients [6] might facilitate such objectives for guaranteeing to patients affected by thalassemia forms or other hemoglobinopathies equal access to quality care in every region of the world by endorsing education, research, awareness, and support. The continued promotion of public awareness campaigns and screening programs might represent a way, as well as always sending all women of childbearing age to thalassemia services, as well as subjects with doubtful haemoglobin and blood count tests, since normal HbA2 values with low MCH often underlie an α-thalassemia trait, and borderline HbA2 values can be associated with the presence of α triplication or a silent β-thalassemia trait [19,20].

The search for the bearer allows young people to acquire and maintain awareness of the disease, and provides at-risk couples with adequate knowledge to make informed decisions about reproductive options, to limit the birth of new cases of the disease only to those derived from choice. Early identification of the patient’s thalassemia and the differential diagnosis between thalassemia major and intermedia allows the prompt treatment of the patient and guarantees them a longer and qualitatively better life. Probably, the clinical application of a new generation of technologies might be of help. The advanced molecular techniques, i.e., targeted sequencing by next-generation sequencing (NGS) and third-generation sequencing (TGS), have been recently suggested as more appropriate and valuable for DNA analysis of thalassemia [26,27]. NGS results are superior at variant calling to TGS thanks to its lower error rates, while the longer-read nature of the TGS permits haplotype-phasing, revealing exceptional variant discovery on the homologous genes. In addition, the constant improvement of these sequencing and bioinformatics will allow precise thalassemia detections. Thus, transitioning laboratories from conventional DNA analysis to NGS or TGS and following the guidelines towards a single assay will contribute to a better diagnostics approach to thalassemia. Another aspect to improve in the management of thalassemia is its treatment. It is well recognized that the conventional treatment of thalassemia patients is essentially based on the correction of anaemia through regular blood transfusions and iron chelation therapy [6]. However, allogeneic hematopoietic stem cell transplantation (HSCT) might represent an available technique with curative potential. However, the diverse frequency and severity of long-term growth and endocrine modifications after conventional treatment, as well as after HSCT, have been described [28]. Consequently, transplantation approaches and careful post-transplantation follow-up of patients have recently been improved for guaranteeing survival outcomes, and HSCT has now been applied in several patients with hemoglobinopathies worldwide, such as patients affected by thalassemia [28,29]. Good current experiences are leading to the recommendation of hematopoietic stem cell transplantation in cases of thalassemia from a matched family or unrelated donor, without secondary organ damage due to transfusion. In the case of patients with sickle cell anaemia, however, transplantation indications require transfusion dependence, and can cause organ damage. Recently, gene therapy has been proposed [30,31,32,33,34], such as the CRISPR/Cas9 system [35]. Studies have shown that the genome of β-thalassemia patients can be modified using the CRISPR/Cas9 technique, and this approach might be promising for the treatment of β-thalassemia [34,35]. Certainly, ulterior studies are imperative. Cotemporally, these improvements lead to another problem, which is the ageing thalassemia population, which will interest the patients living in areas where disease-specific programs offer or will offer access to modern therapies (i.e., those abovementioned) guaranteeing a prolonged survival similar to the normal population. The ageing thalassemia population shows or will have a new spectrum of comorbidities resulting from increasing age, that may expose the advances in prognosis provided by promising new therapies to new challenges in diagnosis, monitoring and treatment. Thus, TIF suggests new efforts to resolve the changing epidemiology and clinical spectrum of patients with β-thalassemia, and to propose actions to be undertaken to address the emerging spectrum of comorbidities resulting from ageing [4].

## Figures and Tables

**Table 1:** Frequencies of the mutations in the analysed genes in the migrants group.

Genetic Variants	Genes	Number of Cases Observed	Frequencies (%) *
*-α3.7 deletion*	*α*	26	51
*−3.7 gene triplication*	*α*	1	2
*-α4.2 deletion*	*α*	1	2
*-α3.7 (homo) deletion*	*α*	1	2
*–SEA deletion*	*α*	1	2
*codon 6 [A > T] HbS*	*β*	3	6
*codon 39 [C > T]*	*β*	1	2
*codon 6 [G > A] HbC*	*β*	1	2
*codon 44 [-C]*	*β*	1	2
*codon 6 [A > T] HbS (homo)*	*β*	3	6
*Codon 39 [C > T]/codone6 [A > T] HbS*	*β*	1	2
*-α3.7 + codone6 [A > T] HbS deletion*	*α + β*	8	16
*-α3.7 + codone6 [A > T] HbS (homo) deletion*	*α + β*	2	4
*-α3.7 + codone6 [G > A] HbC deletion*	*α + β*	1	2

* The single frequencies have been assessed by considering the total of carrier subjects of a mutation in the genes studied.

**Table 2:** Frequencies of the mutations in the groups A, B and C.

Genetic Variants in α Gene	N. of Cases	Frequencies (%)
*-α3.7 deletion*	390	76
*−3.7 gene triplication*	63	12
*α2 IVS 1-5nt*	30	6
*-α3.7 (Homo) deletion*	11	2
*-α4.2 deletion*	5	1
*Codon α_2_INIT cd [T > C]*	3	0.6
*–MED deletion*	2	0.4
*α* *2 cd142 [T > C] Hb Constant Spring*	2	0.4
*α2 cd142 [T > A] Hb Icaria*	1	0.2
*-α3.7/α2 IVS 1-5nt*	1	0.2
*α_2_ polyA-1*	1	0.2
*-α3.7/anti-3.7 gene triplic*	3	0.6
*-α3.7/-MED*	1	0.2
*Genetic variants in the β gene*	N. of cases	Frequencies (%) *
*Codon 39 [C > T]*	94	38
*IVS1.6 [T > C]*	37	15
*IVS1.110 [G > A]*	30	12
*IVS1.1 [G > A]*	28	11
*Codon 6 [A > T] HbS*	12	5
*IVS2.745 [C > G]*	9	4
*IVS2.1 [G > A]*	8	3
*Codon 6 [-A]*	6	2.4
*−101 [C > T]*	4	1.6
*−87 [C > G]*	4	1.6
*IVS1.2 [T > A]*	1	0.4
*Codon 5 [-CT]*	1	0.4
*IVS1.6 [T > C] (homo)*	1	0.4
*Codon 39 [C > T]/IVS1.6 [T > C]*	2	0.8
*Codon 39 [C > T]/IVS1.1 [G > A]*	1	0.4
*Codon 39 [C > T]/IVS1.110 [G > A]*	3	1.2
*Codon 39 [C > T]/codone6 [G > A] HbC*	1	0.4
*Codon 39 [C > T]/−101 [C > T]*	1	0.4
*Codon 6 [G > A] HbC/IVS2.1 [G > A]*	1	0.4
*Codon 39 [C > T]/IVS2.1 [G > A]*	1	0.4
*Genetic variants in the α and β genes*	N. of cases	Frequencies (%) *
*anti-3.7 gene triplic + codone 39 [C > T]*	6	19
*-α3.7 + codone6 [A > T] HbS deletion*	5	16
*-* *α* *3.7 + codone39 [C > T] deletion*	5	16
*-* *α* *3.7 + IVS1.6 [T > C] deletion*	5	16
*-α3.7 + IVS1.1 [G > A] deletion*	2	6
*anti-3.7 gene triplic + IVS2.745 [C > G]*	2	6
*-α3.7 + IVS2.745 [C > G] deletion*	1	3
*anti-3.7 gene triplic + IVS1.6 [T > C]*	1	3
*anti-3.7 gene triplic + IVS1.110 [G > A]*	1	3
*homozygous -α3.7 + IVS2.745 [C > G] deletion*	1	3
*-α3.7 + IVS1.110 [G > A]/ IVS2.1 [G > A] deletion*	1	3
*anti-3.7 gene triplic + −101 [C > T]/IVS1.110 [G > A]*	1	3
*α2 IVS 1-5nt + hetero IVS1.110 [G > A]*	1	3

* The single frequencies have been assessed by considering the total of carrier subjects of a mutation in the α gene.

**Table 3:** Hematological parameters (mean values and Standard Deviation (SD)) observed in subjects with mutations in the α-globin gene.

Genetic Variants in α Gene	Age	N. of Cases	HbA2%	HbF%	Haemoglobin Variants	MCV fL	MCH pg	Hb g/dL	WBC × 10^9^/L	RBC × 10^12^/L	PLT × 10^9^/L	Hct%	RDW-C%
*-α3.7 deletion*	Adults	320	2.48	0.62	no	79	26	13	7.6	5	248	40	14
			sd ± 0.33	ds ± 0.87		sd ± 4.2	sd ± 1.5	sd ± 1.4	sd ± 2.1	sd ± 0.52	sd ± 59	sd ± 4.1	sd ± 1.8
			[1.00–3.40] ^a^	[0.00–9.80] ^a^		[71–88]	[23–29]	[10.4–15.7]	[3.5–11.7]	[4.0–6.1]	[133–362]	[32–48]	[10.6–17.8]
		1	undeterminable	1.2	Hb Lepore 14.8%	74.9	23.5	15.2	10.7	6.48	249	48.5	15.6
	Children	23	2.65	1.15	no	75.5	24.5	12	8.6	4.9	309	37	14.5
			sd ± 0.003	sd ± 0.009		sd ± 4.2	sd ± 1.5	sd ± 0.6	sd ± 2.2	sd ± 0.4	sd ± 93	sd ± 1.9	sd ± 1.1
			[2.00–3.30] ^a^	[0.00–4.40] ^a^		[67–84]	[21.5–27.5]	[10.8–13.2]	[4.2–13]	[4.21–5.62]	[125–492]	[33.4–40.6]	[12.3–16.6]
		1	2.40	0.80	Hb D 32.2%	64	20.8	10.7	4.2	5.16	210	33	18.5
*α2 IVS1 -5nt*	Adults	30	2.54	0.46		76.1	24.7	12.5	7.1	5.07	249	38.5	14.5
			sd ± 0.24	ds ± 0.41		sd ± 4.7	sd ± 1.4	sd ± 1.1	sd ± 1.6	sd ± 0.41	sd ± 49	sd ± 3.6	sd ± 1.3
			[2.20–3.10] ^a^	[0.00–2.10] ^a^		[67–85]	[22–27]	[10.4–14.6]	[4–10.2]	[4.26–5.88]	[153–346]	[31.6–45.5]	[12–17]
*-α4.2 deletion*	Adults	3	2.7	0.63		80.1	26.5	13.8	10.1	5.18	225	41.6	14.5
			sd ± 0.20	sd ± 0.32		sd ± 1.61	sd ± 1.4	sd ± 2.08	sd ± 3.13	sd ± 0.55	sd ± 48	sd ± 5.2	sd ± 1.3
			[2.50–2.90] ^a^	[0.40–1.00] ^a^		[76.9–83.2]	[23.8–29.1]	[9.7–17.8]	[3.9–16.2]	[4.11–6.25]	[132–318]	[31.2–51.9]	[12–16]
	Children	1	2.4	4.4	no	74.7	26	12.3	5.9	4.8	289	35.8	13.9
*α_2_INIT cd [T > C]*	Adults	3	2.33	0.3	no	77.2	24.4	12.9	7.4	5.26	272	40.6	14.2
			sd ± 0.12	sd ± 0.26		sd ± 1.3	sd ± 0.6	sd ± 0.7	sd ± 2.2	sd ± 0.3	sd ± 61	sd ± 1.7	sd ± 0.9
			[2.20–2.40] ^a^	[0.00–0.50] ^a^		[75–80]	[23–26]	[11.5–14.3]	[3–11.7]	[4.7–5.8]	[152–392]	[37–44]	[12.5–16]
*α_2_ polyA-1*	Adults	1	2.70	0.80	no	76.2	24.6	12.1	9.8	4.92	168	37.5	13.8
*α* *2 cd142 [T > C] Hb C.Spring*	Adults	2	2.4	0.45	no	80.5	25.5	14.5	8.4	5.7	260	45.6	14
			sd ± 0.14	sd ± 0.07		sd ± 1.6	sd ± 0.8	sd ± 0.8	sd ± 0.2	sd ± 0.1	sd ± 72	sd ± 0	sd ± 1.3
			[2.30–2.50] ^a^	[0.40–0.50] ^a^		[77–84]	[24–27]	[13–16]	[7.9–8.8]	[5.4–5.9]	[119–401]	[45.6–45.6]	[11.5–16.5]
*α2 cd142 [T > A] Hb Icaria*	Adults	1	2.3	0.6	no	74.3	25	12.4	10.6	4.87	328	36.2	14.4
*anti-3.7 gene triplic*	Adults	50	2.82	0.74	no	82.2	27.3	13	7.6	4.8	252	39	14
			sd ± 0.38	sd ± 0.73		sd ± 6.2	sd ± 2.3	sd ± 1.3	sd ± 1.8	sd ± 0.4	sd ± 59	sd ± 3.6	sd ± 2
			[1.20–3.50] ^a^	[0.00–3.40] ^a^		[70–94]	[23–32]	[10.5–15.5]	[4.1–11]	[3.9–5.6]	[137–366]	[32–46]	[10–18]
	Children	11	2.83	0.98	no	77.8	26	11.7	9	4.6	309	35	14.5
			sd ± 0.003	sd ± 0.010		sd ± 4.4	sd ± 1.7	sd ± 0.9	sd ± 2.5	sd ± 0.3	sd ± 107	sd ± 2.5	sd ± 1.5
			[2.40–3.30] ^a^	[0.00–2.70] ^a^		[69–86]	[22.5–30]	[10–13.4]	[4.2–14]	[4–5.12]	[100–518]	[30.5–40.5]	[11.6–17]
*-α3.7 (homo) deletion*	Adults	9	2.21	0.44	no	71.8	22.6	12.4	8.42	5.51	217	39.5	14.9
			sd ± 0.64	sd ± 0.30		sd ± 4.1	sd ± 1.3	sd ± 1.1	sd ± 2.69	sd ± 0.51	sd ± 64	sd ± 3.4	sd ± 0.9
			[1.10–2.90] ^a^	[0.00–0.80] ^a^		[63.8–79.8]	[20–25.2]	[10.3–14.6]	[3.15–13.7]	[4.51–6.52]	[91–342]	[32.9–46.2]	[13.2–16.7]
	Children	1	2.9	0.6	no	62.2	19.4	11.8	7.8	6.06	322	37.7	14.8
*--MED*	Adults	1	2.4	0.7	no	68.6	22	14	7.8	6.37	208	43.7	15.9
*double heteroz. -α3.7/*	Adults	2	2.5	0.05	no	88.1	27.5	14.6	6.2	4.97	187	43.7	13.3
*anti-3.7 gene triplic*			sd ± 0.14	sd ± 0.07		sd ± 4.3	sd ± 0.7	sd ± 0	sd ± 0.1	sd ± 0.46	sd ± 9	sd ± 1.9	sd ± 2.1
			[2.40–2.60] ^a^	[0.00–0.10] ^a^		[79.6–96.5]	[26–28.9]	[14.6–14.6]	[6–6.3]	[4.06–5.87]	[168–205]	[39.9–47.4]	[9.2–17.3]
	Children	1	2.7	1.1	no	68.1	22	11.8	9.3	5.33	262	36.3	14.4
*double hetero -α3.7/α2 IVS 1-5nt*	Adults	1	2.7	1.1	no	68.9	19.9	11.9	6.8	5.98	126	41.2	26.3
*double hetero -α3.7/--MED*	Adults	1	0.7	undeterminable	Hb H 7.9%	67.7	20	9.3	7.7	4.73	171	32	-

^a^ Range of observed values. MCV, mean corpuscular volume; MCH, mean corpuscular hemoglobin; Hb, hemoglobin; WBC, White Blood Cell; RBC Red Blood Cell; PLT, Platelet; HCT, hematocrit; RDW, Red Cell Distribution Width.

**Table 4:** Hematologic parameters (mean values and Standard Deviation (SD)) observed in subjects with mutation of the β-globin gene.

Genetic Variants in the β Gene	Age	N. of Cases	HbA2%	HbF%	MCV fL	MCH pg	Hb g/dL	WBC × 10^9^/L	RBC × 10^12^/L	PLT × 10^9^/L	Hct%	RDW-C%
*codon 39 [C > T]*	Adults	66	5.55	1.94	63	20	10.5	8.8	5.4	247	33.5	17
			sd ± 0.55	sd ± 1.43	sd ± 4.2	sd ± 1.4	sd ± 1.7	sd ± 2.5	sd ± 0.9	sd ± 84	sd ± 5.5	sd ± 3.1
			[3.70–7.00] ^a^	[0.40–7.70] ^a^	[54–71]	[17–22]	[7–14]	[4–14]	[3.6–7]	[83–412]	[23–44]	[11–23]
	Children	8	5.39	3.38	57.5	18	10.1	9.2	5.6	342	32.1	18
			sd ± 0.010	sd ± 0.023	sd ± 1.7	sd ± 0.7	sd ± 0.8	sd ± 2.7	sd ± 0.51	sd ± 92	sd ± 2.9	sd ± 2.4
			[3.30–6.30] ^a^	[1.50–8.40] ^a^	[54–61]	[16.5–19]	[8.6–11.6]	[3.8–14.5]	[4.59–6.60]	[162–522]	[26.5–37.8]	[13.4–22.6]
*IVS1.1 [G > A]*	Adults	17	4.91	1.65	62	19	10.6	8	5.5	218	34	17
			sd ± 0.70	sd ± 0.95	sd ± 4.6	sd ± 1.7	sd ± 1.6	sd ± 2.4	sd ± 0.8	sd ± 78	sd ± 5	sd ± 2
			[2.90–5.90] ^a^	[0.00–3.40] ^a^	[53–71]	[16–22.5]	[7.5–14]	[3.3–12.7]	[4–7]	[67–371]	[24–44]	[13–21]
	Children	3	4.93	1.47	60.6	18.7	8.9	7.5	4.72	297	28.5	17.9
			sd ± 0.007	sd ± 0.006	sd ± 1.6	sd ± 0.5	sd ± 2.3	sd ± 1.9	sd ± 1.30	sd ± 80	sd ± 7.2	sd ± 8.2
			[4.10–5.40] ^a^	[1.10–2.10] ^a^	[57.5–64]	[18–20]	[4.4–13.4]	[3.8–11.3]	[2.17–7.27]	[140–454]	[14.3–42.7]	[1.9–33.9]
*IVS2.1 [G > A]*	Adults	8	5.53	1.49	62.4	19.3	8.9	6.3	4.63	202	28.6	17.7
			sd ± 0.51	sd ± 0.63	sd ± 4.1	sd ± 1.5	sd ± 0.9	sd ± 1.7	sd ± 0.78	sd ± 73	sd ± 3.3	sd ± 1.8
			[4.90–6.30]^a^	[0.90–2.90]^a^	[54–70]	[16–22]	[7–10.7]	[3–9.6]	[3.10–6.16]	[58–346]	[22.2–35.1]	[14.1–21.3]
*IVS1.2 [T > A]*	Adults	1	5.4	0.8	61	18.9	7.4	9.4	3.93	226	24	15.6
*codon 6 [-A]*	Adults	6	5.17	1.42	56.7	17.2	9.6	7.08	5.5	278	31.3	18.6
			sd ± 0.98	sd ± 0.56	sd ± 4.1	sd ± 2.1	sd ± 1.8	sd ± 1.89	sd ± 0.44	sd ± 106	sd ± 4.5	sd ± 3.2
			[4.10–6.50] ^a^	[1.00–2.50] ^a^	[49–65]	[13–21]	[6–13]	[3.4–10.8]	[4.64–6.36]	[71–486]	[22.4–40.2]	[12–24.8]
*codon 44 [-C]*	Adults	1	5.9	1.4	58.4	18	10	22.9	5.53	177	32.3	19.1
*−101 [C > T]*	Adults	4	3.75	1.75	87.5	28.5	13.2	6.8	4.64	221	40.6	14.3
			sd ± 0.24	sd ± 0.9	sd ± 1.8	sd ± 0.6	sd ± 0.5	sd ± 1.4	sd ± 0.26	sd ± 35	sd ± 1.8	sd ± 0.5
			[3.50–4.00] ^a^	[0.70–2.80] ^a^	[83.9–91.1]	[27.2–29.7]	[12.2–14.3]	[3.9–9.6]	[4.13–5.1]	[153–289]	[37–44]	[13–15]
*−87 [C > G]*	Adults	2	5.4	3.65	71.4	23.5	12.8	11.8	5.4	294	38.9	15.9
			sd ± 0.71	sd ± 1.34	sd ± 7.4	sd ± 2.1	sd ± 1.9	sd ± 1.3	sd ± 0.3	sd ± 135	sd ± 6.2	sd ± 1.7
			[4.90–5.90] ^a^	[2.70–4.60] ^a^	[57–86]	[19–28]	[9–16.5]	[9.1–14.4]	[4.82–6.04]	[30–559]	[26.7–51.1]	[12–19]
	Children	2	5.6	8.3	65.1	22	11.8	11	5.26	511	34.3	16.8
			sd ± 0.004	sd ± 0.006	sd ± 0.1	sd ± 0.6	sd ± 0.6	sd ± 3.5	sd ± 0.33	sd ± 129	sd ± 2.1	sd ± 0.4
			[5.30–5.90] ^a^	[7.90–8.70] ^a^	[64.8–65.4]	[22–22]	[10.5–13]	[4.2–17.8]	[4.62–5.90]	[257–764]	[30.2–38.3]	[16–17.6]
*IVS1.6 [T > C]*	Adults	32	3.81	0.69	68.5	21.9	12.2	7.6	5.57	243	38.1	15.6
			sd ± 0.46	sd ± 0.42	sd ± 3	sd ± 1.3	sd ± 1.2	sd ± 1.8	sd ± 0.51	sd ± 67	sd ± 3.5	sd ± 1.1
			[3.30–5.00] ^a^	[0.10–1.90] ^a^	[63–74]	[19–24]	[9.9–14.5]	[4–11.2]	[4.57–6.58]	[112–374]	[31–45]	[13.4–17.9]
	Children	1	4.5	1.3	71.7	22.5	12	4.8	5.31	268	38.1	17
*IVS1.110 [G > A]*	Adults	20	4.90	1.37	62.5	19.7	11.9	8.2	6.0	267	37.5	16.3
			sd ± 0.66	sd ± 0.88	sd ± 3.6	sd ± 1.1	sd ± 1.4	sd ± 1.9	sd ± 0.6	sd ± 66	sd ± 4.3	sd ± 1.1
			[3.10–5.80] ^a^	[0.60–3.80] ^a^	[55–69.5]	[17.6–21.8]	[9.2–14.6]	[4.4–12]	[4.7–7.2]	[139–396]	[29–46]	[14–18.5]
	Children	7	4.97	1.96	60.1	18.8	10.5	10.5	5.56	310	33.4	16.6
			sd ± 0.008	sd ± 0.01	sd ± 3.5	sd ± 1.1	sd ± 0.8	sd ± 1.9	sd ± 0.51	sd ± 51	sd ± 2.7	sd ± 1.4
			[3.20–5.50] ^a^	[0.70–4.00] ^a^	[53–67]	[17–21]	[9–12]	[6.7–14.2]	[4.57–6.55]	[211–409]	[28.2–38.6]	[13.9–19.4]
*IVS2.745 [C > G]*	Adults	6	4.65	1.65	61.1	18.9	10.9	8.4	5.75	226	35	16.5
			sd ± 0.007	sd ± 0.01	sd ± 5.8	sd ± 2.7	sd ± 2.2	sd ± 2.7	sd ± 1.09	sd ± 93	sd ± 6.7	sd ± 1.5
			[3.80–5.50] ^a^	[0.10–4.00] ^a^	[50–72]	[14–24]	[6.6–15.2]	[3.1–13.7]	[3.62–7.89]	[43–409]	[21.9–48.1]	[13.6–19.4]
	Children	1	2.8	5.4	74.7	25	11.3	5.18	4.55	388	34	15.9

^a^ Range of observed values. MCV, mean corpuscular volume; MCH, mean corpuscular hemoglobin; Hb, hemoglobin; WBC, White Blood Cell; RBC Red Blood Cell; PLT, Platelet; HCT, hematocrit; RDW, Red Cell Distribution Width.

**Table 5:** Hematologic parameters (mean values and Standard Deviation (SD)) observed in subjects were carriers of HbS haemoglobin variant.

Haemoglobin Variant	Age	N. of Cases	HbA2%	HbF%	HbS%	MCV fL	MCH pg	Hb g/dL	WBC × 10^9^/L	RBC × 10^12^/L	PLT × 10^9^/L	Hct%	RDW-C%
codon 6 [A > T] HbS	Adults	9	3.28	0.73	38.6	84	28	13	8	4.6	218	38.5	14
			sd ± 0.004	sd ± 0.003	sd ± 0.012	sd ± 5.4	sd ± 1.3	sd ± 0.6	sd ± 2.2	sd ± 0.3	sd ± 32	sd ± 2.2	sd ± 0.9
			[2.80–3.90] ^a^	[0.30–1.20] ^a^	[36.40–40.4] ^a^	[73–94]	[26–31]	[12–14]	[3.7–12]	[4–5.2]	[156–281]	[34–43]	[12–16]
	Children	2	3.4	1.2	37.55	74.9	24.8	12.5	6.6	5.03	331	37.7	14.7
			sd ± 0.006	sd ± 0.003	sd ± 0.02	sd ± 0.2	sd ± 0.4	sd ± 0.4	sd ± 0.9	sd ± 0.04	sd ± 0.106	sd ± 0.2	sd ± 2.0
			[3.00–3.80] ^a^	[1.00–1.40] ^a^	[36.2–38.9] ^a^	[74.75]	[24.25]	[11.8–13]	[4.7–8.4]	[4.95–5.11]	[331–331]	[37.2–38.1]	[10.8–18.6]

^a^ Range of observed values. MCV, mean corpuscular volume; MCH, mean corpuscular haemoglobin; Hb, haemoglobin; WBC, White Blood Cell; RBC Red Blood Cell; PLT, Platelet; HCT, haematocrit; RDW, Red Cell Distribution Width.

**Table 6:** Hematologic parameters (mean values and Standard Deviation (SD)) observed in subjects with mutation of the α-globin and β-globin genes.

Genetic Variants	Age	N. of Cases	HbA2%	HbF%	Haemoglobin Variants	MCV fL	MCH pg	Hb g/dL	WBC × 10^9^/L	RBC x 10^12^/L	PLT x 10^9^/L	Hct %	RDW-C%
*-α3.7 deletion/cod 39 [C > T]*	Adults	5	5.58	1.6	No	65.5	20.6	12.4	7.9	5.99	191	39.2	15.7
			sd ± 0.27	sd ± 0.75		sd ± 1.4	sd ± 0.5	sd ± 0.5	sd ± 2.4	sd ± 0.27	sd ± 32	sd ± 1.7	sd ± 0.4
			[5.30–5.90] ^a^	[0.80–2.70] ^a^		[62.8–68.3]	[19.7–21.6]	[11.4–13.4]	[3.3–12.6]	[5.45–6.52]	[129–253]	[36–42.5]	[15–16.5]
*-* *α* *3.7 deletion/IVS1.6 [T > C]*	Adults	3	3.87	0.97	No	72.7	22.4	11.1	6.3	5.01	236	36.2	15.4
			sd ± 031	ds ± 0.31		sd ± 6.1	ds ± 3.5	sd ± 0.8	sd ± 1.3	sd ± 0.56	sd ± 22	sd ± 1.2	sd ± 0.3
			[3.60–4.20] ^a^	[0.70–1.30] ^a^		[60.9–84.6]	[15.5–29.3]	[9.6–12.6]	[3.9–8.8]	[3.91–6.10]	[193–279]	[33.8–38.6]	[14.9–15.9]
*-α3.7 deletion/IVS1.1 [G > A]*	Adults	1	5.9	1	No	63.3	21	11.5	10	5.46	236	34.5	14.9
	Children	1	6.7	2.8	no	57.7	19	10.1	16.8	5.31	446	30.6	18.3
*-α3.7 deletion/IVS1.110 [G > A]*	Children	1	5.5	0.6	no	62.5	19.3	11.3	8.7	5.82	213	36.4	15
*-α3.7 deletion/IVS2.745 [C > G]*	Adults	1	5.3	1.1	no	65.9	20.2	12.6	5.6	6.21	260	41	15.6
*-α3.7 deletion/cod 6 [A > T] HbS*	Adults	1	3.6	0.1	34.5	80.5	28	12.6	10	4.55	291	36.6	13.4
	Children	3	3.67	0.13	32.43	73.8	23.6	11.5	8.4	4.88	280	36	14.7
			sd ± 0.12	sd ± 0.23	sd ± 0.87	sd ± 3.7	sd ± 1.6	sd ± 0.8	sd ± 0.8	sd ± 0.06	sd ± 23	sd ± 2.1	sd ± 1.6
			[3.60–3.80] ^a^	[0.00–0.40] ^a^	[30.72–34.15] ^a^	[66.6–81.1]	[20.6–26.7]	[9.9–13.2]	[6.7–10]	[4.76–5.0]	[236–324]	[31.8–40.2]	[11.6–17.8]
*-α3.7 (homoz) deletion/IVS2.745 [C > G]*	Adults	1	2.7	0.6	no	65.7	21	13.1	11.3	6.12	250	40.2	13.8
*α2 IVS 1-5nt/IVS1.110 [G > A]*	Adults	1	4.8	0.7		64	20.4	12.9	5.6	6.32	183	40.5	17.3
*anti-3.7 gene triplic/cod39 [C > T]*	Adults	4	5.73	2.8	no	61.3	19	9.3	6.2	4.9	210	29.6	20.1
			sd ± 0.59	sd ± 1.12		sd ± 4	sd ± 1.2	sd ± 1.4	sd ± 1.6	sd ± 0.8	sd ± 67	sd ± 3.4	sd ± 6.1
			[5.10–6.40] ^a^	[1.80–4.00] ^a^		[53.5–69.1]	[16.5–21.4]	[6.6–12]	[3.1–9.2]	[3.4–6.3]	[77–343]	[23–36.2]	[8.1–32.2]
	Children	2	6.45	1.3	no	57.5	18	8.7	6.9	4.85	356	27.9	19
			sd ± 0.07	sd ± 0.14		sd ± 3.3	sd ± 1.3	sd ± 0.6	sd ± 3.3	sd ± 0.05	sd ± 6	sd ± 1.3	sd ± 1
			[6.40–6.50] ^a^	[1.20–1.40] ^a^		[50.9–64]	[15.3–20.6]	[7.6–9.8]	[0.5–13.3]	[4.75–4.94]	[343–368]	[25.2–30.5]	[17–21]
*anti-3.7 gene triplic/IVS1.110 [G > A]*	Adults	1	2.4	1.3	no	67.2	20	13.2	9.3	6.51	187	43.7	16.7
*anti-3.7 gene triplic/IVS2.745 [C > G]*	Adults	2	5.35	2.4	no	64.4	20	9	11.2	4.49	228	28.8	17.5
			sd ± 0.07	sd ± 1.41		sd ± 3.8	sd ± 1.4	sd ± 1.2	sd ± 5.3	sd ± 0.91	sd ± 30	sd ± 4	sd ± 1.9
			[5.30–5.40] ^a^	[1.40–3.40] ^a^		[56.9–71.9]	[17.2–22.8]	[6.6–11.3]	[0.8–21.5]	[2.72–6.26]	[168–287]	[20.9–36.6]	[13.7–21.2]
*anti-3.7 gene triplic/IVS1.6 [T > C]*	Adults	1	3.7	1	no	64.3	21	11.7	7.32	5.68	268	36.5	15.5

^a^ Range of observed values. MCV, mean corpuscular volume; MCH, mean corpuscular hemoglobin; Hb, hemoglobin; WBC, White Blood Cell; RBC Red Blood Cell; PLT, Platelet; HCT, hematocrit; RDW, Red Cell Distribution Width.

## Data Availability

Not applicable.
